# Lightweight FMCW radar framework for human activity recognition under limited data conditions

**DOI:** 10.1038/s41598-026-44815-8

**Published:** 2026-03-22

**Authors:** Ali Samimi Fard, Mohammadreza Mashhadigholamali, Samaneh Zolfaghari, Hajar Abedi, Mainak Chakraborty, Luigi Borzì, Masoud Daneshtalab, George Shaker

**Affiliations:** 1 Department of Control and Computer Engineering, Polytechnic University of Turin, Turin, Italy; 2 School of Innovation, Design and Engineering, Mälardalen University, Västerås, Sweden; 3 Gold Sentintel Inc, Waterloo, ON Canada; 4 Department of Electrical and Computer Engineering, University of Waterloo, Waterloo, ON Canada

**Keywords:** Human activity recognition, Frequency-modulated continuous wave radar, Deep learning, Ambient assisted living, Remote sensing, Engineering, Mathematics and computing

## Abstract

Human activity recognition (HAR) using frequency-modulated continuous wave (FMCW) millimeter-wave radar is a promising alternative to wearable and vision-based systems due to its unobtrusive and privacy-preserving nature. However, modeling multi-dimensional radar data under limited training samples while remaining robust to user and environmental variations is challenging, particularly for edge-based applications. To address this challenge, we propose a lightweight artificial intelligence-based framework for FMCW radar-based HAR that enables accurate and computationally efficient activity recognition on edge devices. The framework processes radar-derived Range-Doppler, Range-Azimuth, and Range-Elevation feature maps as structured multi-dimensional data vectors rather than conventional two-dimensional images, allowing compact representation of motion dynamics and spatial relationships. A lightweight deep learning architecture combining a modified ResNet-18 with depthwise separable convolutions and a bidirectional long short-term memory module is employed to extract spatial–temporal features with reduced complexity. To improve generalization under limited data conditions, we used data augmentation strategies including spatial shifting, intensity scaling with bias shift, horizontal Doppler flipping, and additive Gaussian noise. The framework is evaluated on a newly collected 60 GHz FMCW radar dataset covering seven daily activities in a realistic home-like environment. Experiments using cross-scene and leave-one-person-out validation demonstrate superior performance over baseline methods, achieving up to 91.98% accuracy and 89.82% F1-score.

## Introduction

Human Activity Recognition (HAR) has become essential for applications in smart homes, healthcare monitoring, gait analysis, and fall detection^1–3^. The rising elderly population has heightened the need for reliable activity monitoring to improve quality of life and provide timely emergency responses^2^. Recent advances in Machine Learning (ML), Deep Learning (DL), and low-cost sensors have made HAR technologies more accessible, enabling continuous, non-intrusive monitoring and quick interventions during emergencies^4,5^. When it comes to sensors, they can be divided into two main categories: wearable and non-wearable^6^.

Despite their widespread adoption, wearable sensors come with several challenges. They can cause discomfort for users, rely heavily on battery life, and often become less reliable during activities like bathing or sleeping^7^. Their accuracy also depends on where they are placed on the body^8^. These issues highlight the need for alternative solutions. In this context, non-wearable sensors offer a different approach by gathering data from the surrounding environment without needing users to carry them^3,9–11^. However, they come with their own set of challenges, such as privacy concerns with video cameras^12^, and limitations from environmental factors (e.g., sunlight, obstructions) for infrared sensors^3,6,13^.

In recent years, radar-based sensing, particularly Frequency-Modulated Continuous Wave (FMCW) radar, has become a promising solution for non-intrusive monitoring without wearable devices or cameras^3,14^. Known for high accuracy and robustness, even in low-light and obstructed environments^12,14,15^, FMCW radars transmit chirp signals and use Doppler shifts to capture detailed motion information. This includes larger movements like walking and sitting, as well as precise actions like picking up objects or falling^3,16^.

Notably, while HAR has made substantial progress through the integration of advanced ML and DL techniques, the availability of large, high-quality datasets remains a persistent challenge. Furthermore, the increasing complexity and computational demands of state-of-the-art models hinder their applicability for real-time and edge processing. Therefore, there remains a need for accurate, lightweight, and privacy-preserving solutions that can operate efficiently on edge devices.

To address these challenges, we propose an Artificial Intelligence (AI)-based HAR framework that directly processes structured radar representations derived from Range–Doppler (RD), Range–Azimuth (RA), and Range–Elevation (RE) measurements. Unlike most existing approaches that convert radar data into 2D image-like representations, these features are treated as three-dimensional (3D) data vectors that preserve the separable physical dimensions of range, angle, and Doppler. This design avoids imposing artificial spatial correlations and translation invariance inherent to image-based convolutional processing, resulting in a more physically meaningful and interpretable representation of human motion.

To effectively capture both spatial characteristics and temporal dynamics of human activities, we design a hybrid DL architecture that integrates a modified ResNet-18 backbone with Bidirectional Long Short-Term Memory (BiLSTM) layers. The ResNet component extracts discriminative spatial features from vectorized radar inputs, while the BiLSTM models temporal dependencies across consecutive radar frames.

To enable real-time operation on resource-constrained devices, depthwise separable convolutions are incorporated into the network architecture. This significantly reduces the number of trainable parameters and computational complexity compared to standard convolutional layers, contributing to a lightweight design suitable for edge and embedded sensing platforms commonly used in indoor environments.

To enhance robustness and generalization, we introduce a set of data augmentation strategies applied directly to radar vectors, including spatial shifting, temporal warping, intensity scaling, and additive noise. These augmentations correspond to realistic variations in target position, motion dynamics, reflectivity, and measurement noise, and differ fundamentally from image-domain augmentation techniques that may distort the physical interpretation of radar signals.

We collect a new dataset in a realistic indoor environment using a low-resolution 60 GHz FMCW millimeter-wave radar. The dataset includes a diverse set of intricate and less-studied human activities, covering both steady-state actions and transitional movements. Unlike many existing datasets, the recordings reflect real-life indoor sensing conditions and are designed to evaluate generalization when only a small number of subjects are available.

The proposed framework is evaluated using multiple cross-validation strategies, including cross-subject and cross-scene evaluations, and is compared against representative ML and DL baselines.

The remainder of this paper is organized as follows. The ‘Related work’ section reviews existing studies on radar-based HAR and highlights their limitations. ‘Methodology’ describes the proposed framework, including the radar setup, data acquisition process, and feature extraction methods. ‘Experimental evaluation’ presents the experimental setup, results, and comparative performance analysis. ‘Discussion’ interprets the findings, emphasizing the key contributions and potential applications. Finally, ‘Conclusion’ summarizes the study and outlines directions for future research.

## Related work

Recent advancements in HAR systems have leveraged sophisticated DL models and innovative feature extraction techniques to enhance accuracy and efficiency^17^. A significant number of prior studies have adopted a transfer learning paradigm, converting raw radar returns into 2D image representations to leverage pre-trained Computer Vision (CV) architectures^12^. In addition, due to the scarcity of benchmark datasets available for evaluation, many studies have relied on the University of Glasgow dataset^18^ as a primary evaluation platform.

Abdu et al.^12^ employed micro-Doppler spectrogram images extracted from this benchmark and compared three different pre-trained models using a transfer learning approach. Structurally, they treated these spectrograms as two-dimensional (2D) RGB images, enabling the use of deep CNNs such as AlexNet and VGG-19 to extract features from visual patterns. They incorporated a channel attention module to improve performance in classifying six activities, achieving 99.77% accuracy through a combination of these models, enhanced by Canonical Correlation Analysis (CCA) feature fusion and a Support Vector Machine (SVM) classifier. However, by relying on an image-based representation, the model assumes spatial correlations and translation invariance between pixels that may not reflect the physical nature of radar signals. Despite this promising performance, the dual-Convolutional Neural Network (CNN) fusion framework introduces substantial computational complexity that may hinder real-time deployment.

Kim et al.^19^ proposed a framework that combines Range-Time-Doppler (RTD) maps with a Range-Distributed Convolutional Neural Network (RD-CNN), achieving an accuracy of 96.49% in familiar environments using the public University of Glasgow dataset. Their structural approach involves decomposing radar data into specific range-time and Doppler-time planes for processing by a multi-stream CNN. While this effectively captures signatures at different ranges, it still treats these feature maps as image-like inputs, which can impose artificial spatial dependencies on the physical movement data. Their method is also computationally heavier than single-CNN models due to the multiple processing streams required for the decomposed maps, and its suitability for real-time deployment requires further analysis.

More recently, Ayaz et al.^20^ investigated the impact of radar signal preprocessing methods on DL models. They evaluated three 2D representations—Time-Range (TR) maps, Time-Doppler (TD) spectrograms, and Smoothed Pseudo-Wigner–Ville Distribution (SPWVD) maps–across four state-of-the-art CNN architectures (VGG-16, VGG-19, ResNet-50, and MobileNetV2) using transfer learning. Structurally, their approach relies on converting raw radar returns into visual time-frequency heatmaps, which are then processed as static images by standard 2D CNN kernels. Their findings reveal a critical trade-off between recognition accuracy and computational efficiency for real-time and edge computing scenarios. The Short-Time Fourier Transform (STFT) combined with MobileNetV2 offers the best balance, achieving 96.3% accuracy with efficient computation, whereas SPWVD yields higher accuracy (98.0%) but requires extensive preprocessing. However, treating radar signatures as 2D pixels imposes artificial spatial correlations that do not inherently account for the multidimensional physical dependencies between range and Doppler. Although the study demonstrated excellent accuracy, it relied solely on the University of Glasgow dataset, which may limit its generalizability across different environments, activity types, and participant populations.

Kruse et al.^21^ proposed a radar point cloud processing pipeline for continuous HAR using Point Transformer networks. Their structural approach converts Single-Input Single-Output (SISO) radar data into Range-Time-Doppler (RTD) point clouds and leverages multi-radar sensor fusion. Instead of processing dense 2D images, they represent human motion as a set of discrete, sparse points in space, using self-attention mechanisms to learn global and local dependencies from the point cloud geometry. The proposed method outperformed CNN-Recurrent Neural Network (RNN) and ResNet-based baselines on a public dataset^22^ with 14 subjects and 9 activities, achieving 86.9% accuracy under a leave-one-person-out validation scheme. Although promising, this dataset was collected in a controlled laboratory environment that does not fully reflect real-world settings. Additionally, the complex structural design of Transformer-based models, which requires significant computational resources to calculate attention maps across large point sets, may constrain their feasibility for real-time deployment on edge devices.

Some researchers have created custom datasets tailored to their specific research goals. The radars used in these works vary in configuration, including operating frequency, maximum detection range, range resolution, and power consumption. Ding et al.^23^ utilized dynamic range-Doppler frames (DRDF) and Spatio-Temporal Convolutional Long Short-Term Memory (ST-ConvLSTM) networks, achieving 96.5% accuracy in classifying 6 movements performed by 16 subjects. Structurally, their framework processes radar data as a sequence of dynamic Range-Doppler frames, where the ST-ConvLSTM architecture simultaneously extracts spatial features and temporal dependencies via convolutional structures within LSTM cells. While this approach effectively models the localized motion intensity within the 2D RD plane, it treats each frame as a grid-based spatial input, relying on the assumption of local spatial continuity within the Doppler-range image. Although computationally efficient, the approach relies on a laboratory-recorded dataset, which may limit its generalization to more complex real-world scenarios.

Bhavanasi et al.^24^ evaluated various ML algorithms on micro-Doppler and RD maps derived from radar data collected in hospital environments. Structurally, their approach utilizes a 3D CNN architecture that treats sequences of 2D Range-Doppler and micro-Doppler heatmaps as volume-based image inputs to capture spatial-temporal patterns. Their CNN models outperformed other algorithms, achieving high accuracy in classifying ten activities performed by 29 subjects. However, by processing these maps as dense image tensors, the model relies on the assumption of spatial correlation between adjacent pixels in the Doppler and range bins, which may not always align with the independent physical properties of the target’s motion. Consequently, the models showed limited cross-environment generalization and high computational requirements, posing challenges for real-time healthcare applications.

Table 1 provides a comprehensive summary of previous studies in the radar-based HAR domain, highlighting the diversity of approaches, datasets, and performance metrics.

**Table 1:** Summary of related works in radar-based human activity recognition.

Ref.	Year	Dataset	# Classes	# Subjects	Radar data domain	Classification algorithm	Best accuracy (%)
^12^	2022	University of Glasgow	6	99	MD spectrogram images	AlexNet + VGG-19 (CCA + SVM)	99.77
^19^	2022	University of Glasgow	6	60	RTD maps	RD-CNN	96.49
^20^	2025	University of Glasgow	6	81	SPWVD	VGG-19	98.01
^21^	2024	Public dataset^22^	9	14	RD–T point clouds	Point transformer	86.9
^23^	2022	Private dataset	6	16	DRDF	ST-ConvLSTM + attention	96.5
^24^	2022	Homelab + Hospital	10	29	RD, MD	CNN	95.0

*MD* Micro-Doppler, *RTD* Range-time-Doppler, *RD* Range-Doppler, *RD-T* Range-Doppler-time, *SPWVD* smoothed pseudo-Wigner-Ville distribution, *DRDF* dynamic range-Doppler frames, *ST-ConvLSTM* spatio-temporal convolutional long short-term memory, *CCA* canonical correlation analysis, *SVM* support vector machine, *CNN* convolutional neural network.

Despite significant progress, most radar-based HAR systems rely on converting radar data into image-based representations such as spectrograms or heatmaps^7,12,19,25^. However, these approaches impose structural constraints that are often incongruent with the physics of radar sensing: (1) limited interpretability due to high visual similarity across different activity patterns, (2) susceptibility to noise and artifacts that degrade image quality and interpretability^26,27^, and (3) the need for large labeled datasets to train DL models effectively^19^.

Standard 2D CNNs further rely on weight sharing and pooling to enforce translation invariance, implicitly assuming that a feature’s semantic meaning is independent of its spatial location (e.g., a visual texture represents the same object whether it appears in the top-left or bottom-right of an image). In radar signal processing, this assumption is physically invalid because the axes of radar maps correspond to absolute physical quantities (range and velocity). Enforcing translation invariance in this domain therefore incorrectly encourages the model to disregard absolute range and velocity information, which is critical for distinguishing targets (e.g., a static versus moving object)^28^.

Furthermore, real-world data collection remains challenging. Many existing studies rely on controlled laboratory environments that do not accurately reflect the complexity and variability of real-life conditions^23^. In clinical settings, deploying HAR systems at scale would require installing numerous radars and processing substantial amounts of data within constrained time frames. This necessitates lightweight models capable of efficient operation across large-scale implementations.

To address these limitations, we propose an alternative approach that directly utilizes raw multi-dimensional radar feature maps—RD, RA, and RE—as input to DL models. Rather than converting these into visual representations, we preserve them as three-channel structured data vectors inputs that retain both spatial and temporal characteristics. This representation enhances the model’s ability to capture complex activity patterns while maintaining interpretability through direct access to physical radar measurements. Applying isotropic 2D filters conflates these distinct physical dimensions, ignoring the absolute positional information critical for accurate trajectory and motion interpretation.

Our validation across various conventional and modern architectures demonstrates robust performance using data from only three participants, thereby reducing the dependency on large-scale datasets that plague image-based approaches. To advance radar-based HAR research with new sensing technologies and benchmark datasets, this study introduces a novel dataset collected using a low-resolution $60\,\text {GHz}$ mmWave FMCW radar, along with a comprehensive framework for data pre- and post-processing in HAR applications. The lightweight nature of our approach makes it particularly suitable for large-scale clinical deployments where computational efficiency is paramount.

## Methodology

**Fig. 1:**
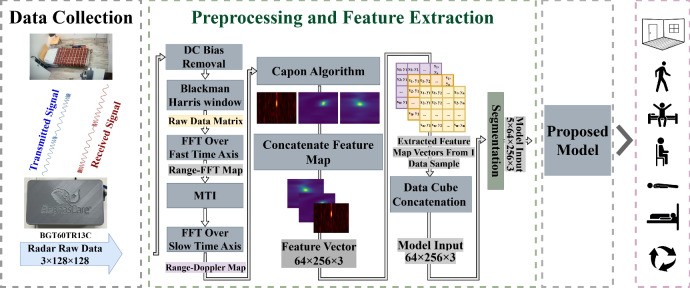
Overview of the proposed lightweight framework for FMCW radar-based HAR.

This section provides an overview of the functional prototype of our proposed framework, which is depicted in Fig. 1. Each component is described in detail in the following subsections.

### Radar setup and data collection

**Fig. 2:**
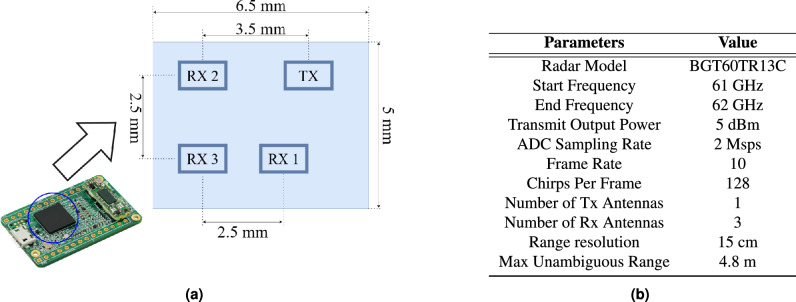
(**a**) Top view and antenna arrangement of the Infineon BGT60TR13C radar sensor. (**b**) Radar configuration parameters and specifications.

This study uses the BGT60TR13C, an FMCW radar system from Infineon Technologies AG^29^. It has a single transmitter and three receivers, operating in the 58-63.5 GHz band with a configurable chirp duration. The antennas are in an L-shaped configuration, with RX1 and RX3 for azimuth and RX2 and RX3 for elevation angle measurements^30^. Figure 2a shows antennas arrangement in the BGT60TR13C radar and Fig. 2b lists the radar configuration parameters used in this study.

**Fig. 3:**
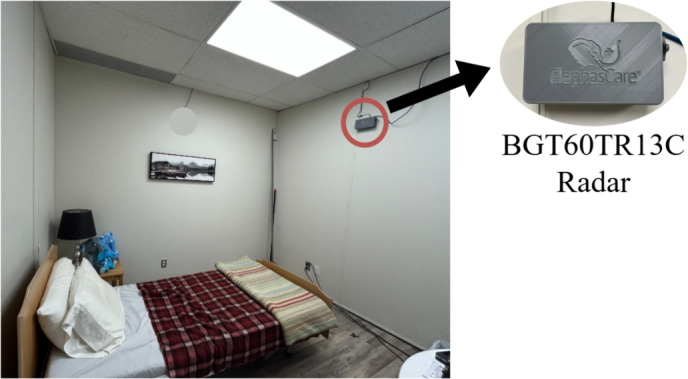
Room layout and radar placement in the home-like data collection environment.

To collect data, the radar-based sensor is arranged to ensure optimal bedroom coverage and maximizing performance. It was installed at a height of 210 cm and angled downward at $30^\circ$. Three healthy subjects participated in the study, performing activities over 16 scenes (recording sessions). Two subjects performed six scenes each, while the third subject completed four scenes. Each scene started with 1 min of data collection in an empty room. Participants then performed the following activities: walking for 2 min, sitting on a bed for 2 min, lying on the bed for 5 min, and lying on the floor for 5 min. In some sessions, they also sat on a chair for 2 min. Data were collected continuously to capture steady-state activities, dynamic activities, and transitions between states. All transitions were grouped into a single “Transition” class to address classification challenges and balance the dataset. Ultimately, data were categorized into seven activity classes: Empty Room, Walking, Sitting on the Bed, Sitting on a Chair, Lying on the Bed, Lying on the Floor, and Transition. Figure 3 illustrates the room layout and radar installation.

### Data preprocessing and feature extraction

In FMCW radar systems, chirp sequences are transmitted via the $TX$ antenna, with reflections captured by $RX$ antennas. The received data forms a three-dimensional array of dimensions $C \times N \times M$, where $C$ represents the number of channels, $N$ denotes the number of chirps per frame, and $M$ indicates the number of samples per chirp. The data structure comprises *fast time* rows (single chirp/range bin data) and *slow time* columns (same-sample data across chirps)^31^.

#### Blackman–Harris window

The preprocessing pipeline involves DC bias offset removal to eliminate low-frequency noise and artifacts^32^. Subsequently, a Blackman-Harris window function is applied to mitigate spectral leakage in the frequency domain. This windowing technique gradually attenuates signal amplitudes at the boundaries, reducing abrupt transitions that could introduce spurious frequency components during Fourier transformation. This approach enhances the fidelity of spectral analysis, particularly when employing the Fast Fourier Transform (FFT).

#### Range-FFT map

Range detection is performed by computing the FFT along the fast-time axis, where spectral peaks correspond to target distances^33^. The resulting Range-FFT map provides a frequency-domain representation of target reflections. The radar transmits consecutive chirps separated by a fixed time interval to estimate target velocity. Each reflected chirp undergoes a Range-FFT to determine the target position. While the peaks in the Range-FFT spectrum align for both chirps, their phases differ due to target movement. This phase shift provides information about the velocity of the target^34,35^.

#### Moving target indicator (MTI)

The signal consists of two main types of reflections. The first is clutter, which refers to echoes from stationary objects in the environment. The second type originates from moving objects, particularly individuals engaged in daily activities. A clutter removal algorithm is employed to reduce the impact of clutter.

The MTI implements linear filtering to suppress clutter while preserving dynamic target signatures. In FMCW radar systems, the Finite Impulse Response (FIR) implementation offers a good balance of simplicity and effectiveness^36^. At each time step, the maximum absolute value across the slow time dimension for each range bin is denoted as $r_{i,max}$. The MTI filter output $t_i$ is then calculated as a weighted average of this peak value and the previous filter output $t_{i-1}$, using a weighting factor $\alpha$:

1 $$\begin{aligned} t_i = \alpha \cdot r_{i,max} + (1 - \alpha ) \cdot t_{i-1} \end{aligned}$$

At the initial time step ($t_0$), the filter output $t_i$ is initialized to zero. For each range bin, the MTI filter removes the influence of stationary objects by subtracting $t_{i-1}$ from $r_{i,max}$, resulting in the filtered FFT value $r_{i,filt}$^37^:

2 $$\begin{aligned} r_{i,filt} = \left| r_{i,max} - t_{i-1} \right| \end{aligned}$$

This method of subtracting an estimate of stationary background clutter effectively eliminates static targets while having minimal effect on slow-moving objects. FIR MTI filters are preferred for their simple design, adjustable parameters, and linear phase response^36,37^.

#### Range-Doppler map

**Fig. 4:**
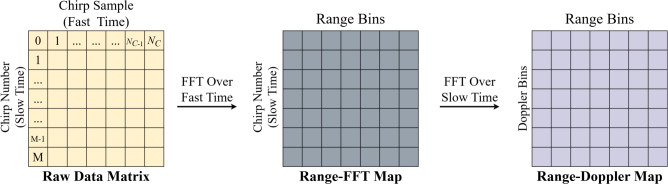
Range-Doppler map data processing.

The processing sequence continues with a second FFT applied along the vertical axis to extract Doppler information for each channel. The output is the RD map. Figure 4 illustrates range-FFT and RD map.

#### Capon algorithm

Accurate three-dimensional localization with FMCW MIMO radar requires reliable estimation of range, Doppler, and angle of arrival (AoA). In this work, we apply the Capon beamformer (minimum-variance distortionless response, MVDR) per RD bin across the antenna array to obtain high-resolution AoA estimates in the presence of noise and interference. Scanning the Capon spectrum over candidate angles for each $(R,f_D)$ cell yields RA maps (and RE maps if elevation channels are available).

Assuming up-chirps and a single point target for clarity, for the transmitted signal $s(t)$, the received baseband signal at the $l{\text {th}}$ receive element is

3 $$\begin{aligned} x_l(t_f,t_s) \;=\; b_l\ \cdot e^{-j\!\Big ( 2\pi f_b\ \;+\; \tfrac{2v}{\lambda _{\max }}\, t_s \;+\; \tau _l \;+\; \alpha _l \;+\; \Delta \psi _l(t_f,t_s) \Big )} \;+\; e_l(t_f,t_s), \end{aligned}$$

where $t_f$ is the *fast time* (sample index within a chirp), $t_s$ is the *slow time* (chirp/frame index), $f_b$ is the beat frequency, $v$ is the radial velocity, and $\lambda _{\max }$ is the wavelength at the start frequency. The terms $b_l$ and $\alpha _l$ model channel-dependent magnitude and phase mismatch, $\tau _l$ is the AoA-dependent phase at the $l^{\text {th}}$ receiver, $\Delta \psi _l(t_f,t_s)$ is residual phase noise, and $e_l(t_f,t_s)$ is additive noise.

For a stationary target (Doppler omitted), the beat frequency is

4 $$\begin{aligned} f_b \;=\; S\,\frac{2\,d}{c}, \end{aligned}$$

with $S$ the chirp slope, $d$ the target range, and $c$ the speed of light.

Stacking all $L$ receiver channels into a vector gives

5 $$\begin{aligned} x_l(t_f,t_s) \;=\; {\Gamma }\ \cdot a(\theta ) \cdot y(v,f_b,t_f,t_s) \cdot s(t_f,t_s) \;+\; e_l(t_f,t_s) \end{aligned}$$

with

$${\Gamma } \!=\! \begin{pmatrix} b_1 \cdot e^{-j\alpha _1} & & 0 \\ & \ddots & \\ 0 & & b_L \cdot e^{-j\alpha _L} \end{pmatrix},\quad {\bf a}(\theta ) \!=\! \begin{pmatrix} e^{-j\tau _1}\\ \vdots \\ e^{-j\tau _L} \end{pmatrix},\quad y(v,f_b,t_f,t_s) \!=\! e^{-j\!\left( 2\pi f_b \cdot t_s + \tfrac{2v}{\lambda _{\max }} t_s + \Delta \psi (t_f,t_s)\right) }.$$

For $K$ targets at possibly different ranges/angles,

6 $$\begin{aligned} x_l(t_f,t_s) \;=\; {\Gamma }\ \cdot A({\theta })\ \cdot Y(v,f_b,t_f,t_s) \;+\; e_l(t_f,t_s) \end{aligned}$$

where $A\in \mathbb {C}^{L\times K}$ collects the steering vectors, and $Y$ is diagonal with entries from $y(v,f_b,t_f,t_s)$.

Assuming the additive noise is uncorrelated with ${\bf Y}$, the spatial covariance is

7 $$\begin{aligned} {R} \;=\; {E}\{{x} \cdot {x}^H\} \;=\; P_s\ \cdot {\Gamma }\ \cdot {A}({\theta }) \cdot {A}^H({\theta })\ \cdot {\Gamma }^H \;+\; {R}_n \end{aligned}$$

where $P_s$ is the signal power and ${R}_n$ is the (positive definite) noise covariance. The beamformer output power for weights ${w}$ is

8 $$\begin{aligned} \Phi \;=\; {w}^H {R}\,{w}. \end{aligned}$$

The Capon problem minimizes output power subject to unit response in the look direction $\theta _0$:

9 $$\begin{aligned} \min _{{w}} \;{w}^H {R}\,{w} \quad \text {s.t.}\quad {w}^H {a}(\theta _0)=1. \end{aligned}$$

Using a Lagrange multiplier,

10 $$\begin{aligned} \mathscr {L}({w},\lambda ) = {w}^H {R}\,{w} - \lambda \left( {w}^H {a} - 1\right) , \end{aligned}$$

yields the well-known solution

11 $$\begin{aligned} {w}(\theta _0) \;=\; \frac{{R}^{-1}{a}(\theta _0)}{{a}^H(\theta _0)\,{R}^{-1}{a}(\theta _0)}. \end{aligned}$$

The MVDR spatial spectrum evaluated at a test angle $\hat{\theta }$ is

12 $$\begin{aligned} \Phi (\hat{\theta }) \;=\; {w}^H(\hat{\theta })\, {R}\, {w}(\hat{\theta }) \;=\; \frac{1}{{a}^H(\hat{\theta })\, {R}^{-1}\, {a}(\hat{\theta })}. \end{aligned}$$

Scanning $\Phi (\hat{\theta })$ over angle(s) for each RD bin produces RA (and RE) heat maps for subsequent localization and mapping. For more details on the Capon beamforming algorithm, refer to our previous work^38^.

The final 3D data structure, called the data cube, is formed by combining the RD, RA, and RE feature maps. Before feeding data to DL models, segmentation is done on sets of five consecutive data cubes (corresponding to 0.5 second) to balance granularity and efficiency. If all five data cubes have the same label activity, they are merged into a single segment. If activities differ, such as three matching and two different, the matching cubes are excluded, and segmentation pauses. It resumes with the next group of data cubes for the subsequent activity, ensuring consistency and preventing conflicting labels from merging.

**Fig. 5:**
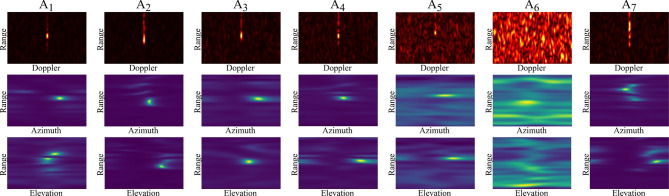
Feature maps corresponding to different activities: ($A_1$) walking, ($A_2$) sitting on the bed, ($A_3$) sitting on the chair, ($A_4$) lying down on the bed, ($A_5$) lying down on the floor, ($A_6$) empty room, ($A_7$) transition.

Figure 5 illustrates examples of feature maps corresponding to various activities. As shown, the feature maps for dynamic activities exhibit significantly less clutter and noise, allowing us to clearly observe motion-related patterns (Fig. 5, $A_1$ to $A_5$). This clarity is achieved through our implementation of the MTI method. In contrast, the feature map for the ‘Empty Room’ (Fig. 5, $A_6$) shows noticeable clutter and noise, which are echoes from stationary objects.

Converting feature maps into 2D image representations enables the use of conventional transfer learning approaches, but it introduces additional preprocessing and may distort the physical meaning of radar axes. In contrast, the proposed vector-based representation reduces computational overhead on the host processor by skipping the image generation and resizing pipeline, thereby directly addressing latency constraints in real-time monitoring. Moreover, the vector inputs preserve the direct physical mapping of range, velocity, and angle, allowing the network to learn physically meaningful spatio-temporal relationships rather than relying on visual textures. Finally, compact structured tensors combined with depthwise separable convolutions reduce inference latency and memory footprint, making the framework suitable for embedded deployment in edge scenarios.

### Activity recognition models

To provide a comprehensive performance benchmark, several baseline models were implemented and evaluated against our proposed model. Specifically, two conventional ML classifiers—SVM and Multi-Layer Perceptron (MLP)—were employed for activity recognition. In addition, three DL architectures—CNN, BiLSTM, and Convolutional Long Short-Term Memory (ConvLSTM)—were implemented to assess comparative performance further. The configurations of these models are summarized below, with any unspecified parameters set to their default values.

SVM^39^: the kernel was set to a radial basis function (RBF) with regularization term $C=10$ and probability=True.

MLP^40^: the network consisted of two hidden layers with 128 and 64 neurons, and Rectified Linear Unit (ReLU) activation function. Training was performed using the Adaptive moment estimation (Adam) optimizer with a learning rate of $1\times 10^{-3}$, and a maximum of 300 iterations.

**Fig. 6:**
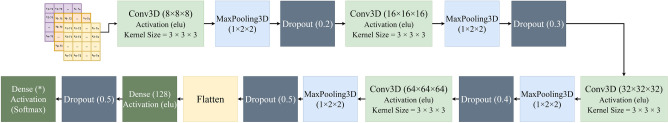
Architecture of the 3D CNN model implemented for activity classification.

CNN^24^: the model consists of four 3D convolutional blocks with 8, 16, 32, and 64 filters respectively, each using a kernel size of ($3 \times 3 \times 3$) and Exponential Linear Unit (ELU) activation function. Each block is followed by a $1 \times 2 \times 2$ MaxPooling layer. The network includes two fully connected layers: one with 128 neurons and another with 7, 6, 5, or 4 neurons (corresponding to the number of activities being classified). Dropout^41^ is applied after each layer at progressively increasing rates from 0.2 to 0.5, excluding the output layer. The network is trained using the Adam optimizer with a cross-entropy loss function, a learning rate of $1 \times 10^{-3}$, and a maximum of 100 epochs with early stopping^42^ (patience = 10 epochs). The model architecture is illustrated in Fig. 6.

**Fig. 7:**
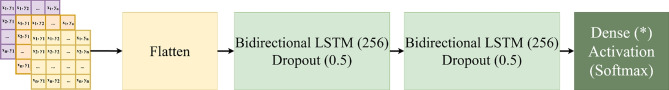
Architecture of the BiLSTM model implemented for activity classification.

BiLSTM^1^: the architecture comprised two BiLSTM layers with 256 units and a dropout of 0.5. The network includes one fully connected layer with 7, 6, 5, or 4 neurons (corresponding to the number of activities being classified). The model was trained using the Adam optimizer with a decay factor of 0.9 and an initial learning rate of $1 \times 10^{-3}$. This learning rate was reduced to 10% of its initial value at the 200th epoch, with training continuing for a maximum of 400 epochs with early stopping (patience = 40 epochs). The model architecture is illustrated in Fig. 7.

**Fig. 8:**
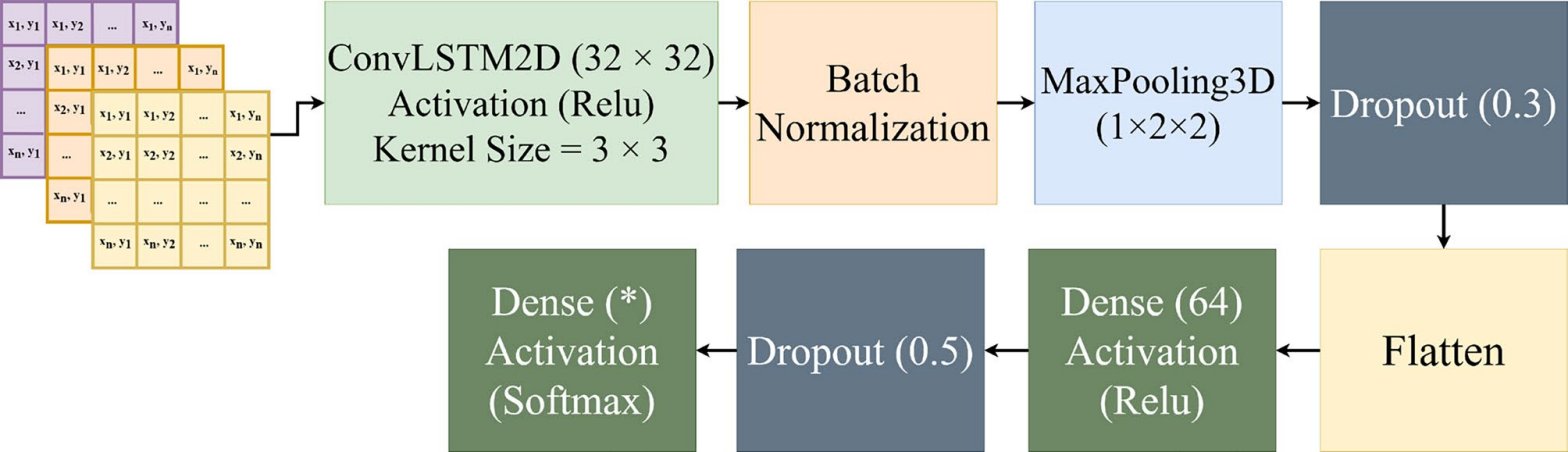
Architecture of the ConvLSTM model implemented for activity classification.

ConvLSTM^43^: the architecture consists of one ConvLSTM block with 32 filters, kernel size of ($3 \times 3$), and ReLU activation function. This is followed by batch normalization^44^, 3D MaxPooling ($1 \times 2 \times 2$), and dropout (0.3). The output is connected to a fully connected layer with 64 neurons and ReLU activation, followed by dropout (0.5). The final fully connected layer contains 7, 6, 5, or 4 neurons (corresponding to the number of activities being classified) with softmax activation. The model was trained using Stochastic Gradient Descent (SGD) with momentum 0.9 and weight decay $1 \times 10^{-4}$, optimizing the categorical cross-entropy loss function. Training utilized a learning rate of $1 \times 10^{-4}$, batch size of 64, and a maximum of 100 epochs with early stopping (patience = 10 epochs). The model architecture is illustrated in Fig. 8.

Proposed lightweight ResNet-18 + BiLSTM: in this study, a hybrid DL model was developed that combines a CNN model for spatial feature extraction with a BiLSTM network for temporal sequence modeling. This hybrid design enables efficient learning from spatial-temporal data such as structured time-sequential vectors.

The CNN backbone is derived from the ResNet-18 architecture^45^, but was modified to improve computational efficiency and reduce the number of parameters. To achieve this, all standard Conv2D layers were replaced with SeparableConv2D, which decompose a standard convolution into depthwise and pointwise operations^46,47^. This factorization significantly reduces computational complexity while maintaining strong feature extraction capability.

The proposed model begins with two separable convolutional layers using 64 filters with kernel sizes of $5\times 5$ and $7\times 7$, each followed by batch normalization, a ReLU activation, and MaxPooling with a stride of 2. Subsequently, three residual stages are applied: two blocks with 64 filters, two blocks with 128 filters (including one downsampling block), and two blocks with 256 filters (including one downsampling block). Each residual block consists of two SeparableConv2D layers, each followed by batch normalization and a ReLU activation. A skip connection adds the input feature map to the block output, enabling residual learning that preserves gradient flow and stabilizes training. Two types of residual blocks are employed. In the standard configuration (down_sample = False), the spatial resolution and number of channels are preserved, allowing identity mapping via the skip path. In the downsampling configuration (down_sample = True), the first convolutional layer uses a stride of 2 to reduce the spatial dimensions and increase the number of output channels. To match dimensions before addition, a $1\times 1$ separable convolution followed by batch normalization is applied to the shortcut path. This design allows the network to progressively reduce spatial resolution while deepening the feature hierarchy, maintaining both efficiency and representational capacity. A global average pooling layer aggregates the final feature maps, followed by a dropout layer with a rate of 0.3 for regularization.

To enable temporal modeling, the CNN is wrapped in a TimeDistributed layer, ensuring shared weights across all five sequential frames. The extracted frame-level feature vectors are then passed to a BiLSTM layer with 128 hidden units, which captures forward and backward temporal dependencies across the frame sequence. A dropout rate of 0.5 is applied within the BiLSTM to prevent overfitting and improve generalization.

The BiLSTM output is fed into two dense layers for feature fusion and classification. The first dense layer contains 128 neurons, followed by ReLU activation, batch normalization, *L2* regularization, and a dropout rate of 0.5. The second dense layer has 64 neurons, also with ReLU activation, batch normalization, and a dropout rate of 0.5. Finally, a dense output layer with 7, 6, 5, or 4 neurons (corresponding to the number of activities being classified) and a softmax activation performs multiclass classification.

To accelerate the training process and mitigate the vanishing gradient problem, kernel weights were initialized using two complementary strategies: the *He normal* initialization^48^ for convolutional layers in the early stages to maintain stable variance with ReLU activations, and the *Xavier uniform* (Glorot) initialization^49^ for dense and residual layers to balance the variance of inputs and outputs. All bias terms were initialized to zero, as recommended in^50^. For optimization, the SGD optimizer was employed with a learning rate of 0.001, a momentum of 0.9, and a weight decay of $1\times 10^{-4}$, with categorical cross-entropy loss.

Several data augmentation techniques were employed during the training phase to mitigate overfitting on limited data, enhance the model’s robustness and generalization to unseen environments and subjects. First, inspired by the physically interpretable range and angle transformation approach proposed by Fusco et al.^51^, small random spatial shifts were applied along the range and Doppler axes to emulate variations in target position and radial velocity. A random two-dimensional circular shift was performed along the range, Doppler, azimuth, or elevation axes with a probability of 0.8 to simulate slight target displacements or variations in radial velocity and angles.

Second, intensity scaling and biasing were applied with a probability of 0.8, where each radar frame was multiplied by a random factor between 0.9 and 1.1 and shifted by a small bias uniformly sampled from $[-0.05, 0.05]$. This operation emulates realistic fluctuations in radar reflectivity and signal strength, as well as variations in radar cross section (RCS), receiver gain, or environmental reflections.

Furthermore, horizontal flipping was applied with a probability of 0.5 to mirror the Doppler spectrum, effectively representing opposite motion directions (e.g., leftward vs. rightward walking).

Finally, as suggested by previous studies^52^, corrupting the training dataset with random noise can effectively improve model robustness. Accordingly, additive Gaussian noise with a mean of 0 and a standard deviation of 0.02 was introduced with probability 0.8 to simulate environmental noise and fluctuations in signal-to-noise ratio (SNR).

**Fig. 9:**
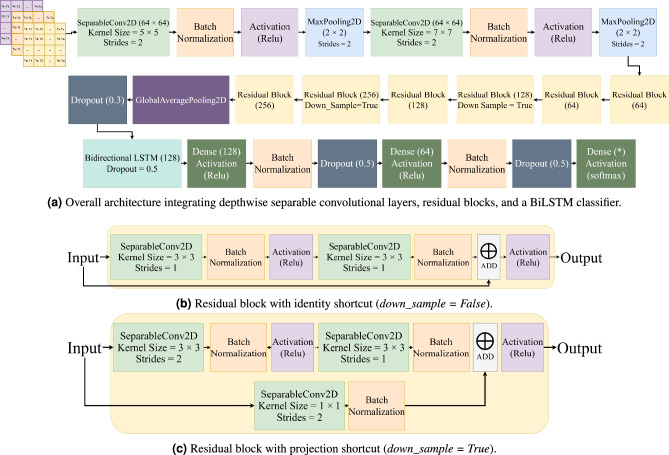
Overview of the proposed lightweight FMCW radar-based HAR framework.

The model was trained for 300 epochs with a batch size of 8, and an early stopping mechanism with a patience of 30 epochs was applied to prevent overfitting. The best-performing model based on validation loss was automatically saved during training. The overall architecture of the proposed model is illustrated in Fig. 9.

The hyperparameters of our proposed framework were determined via a systematic grid search to balance computational efficiency and model performance. The models employed preprocessed RD, RA, and RE data from the FMCW radar as input features. Also, to accelerate training and reduce overfitting, we applied principal component analysis (PCA) with 100 components before feeding the data to the ML baselines models. Our framework processes the spatiotemporal characteristics of FMCW radar outputs through 3D input tensors.

## Experimental evaluation

This section presents experimental results obtained from an FMCW radar dataset, including analyses of feature maps, activities, and ML and DL models. We detail the setup, results, and performance analysis.

### Experimental setup

To validate our approach, we employed two distinct strategies. In the first strategy, Cross-Scene Validation (CSV), we used $80\%$ of the data from 14 of the 16 distinct scenes for training and reserved the remaining $20\%$ for validation. The two remaining scenes were held out for testing. In the second strategy, Leave-One-Person-Out Cross-Validation (LOPO-CV), we repeated the procedure three times—each time leaving one subject out—and computed the accuracy and $F_1$-score. Specifically, in each fold, we trained the model using $80\%$ of the data from the two subjects, reserved the remaining $20\%$ for validation, and used the unseen subject’s data for testing. For example, in the first iteration, the data of the first subject was held out for testing, while data from the second and third subjects was split for training and validation; in the subsequent iterations, the roles were rotated accordingly. Tables 2 and 3 summarize the data distribution for each validation strategy.

**Table 2:** Activity sample counts under CSV approach.

Activity	Training	Validation	Testing	Total
Walking ($A_1$)	6495	1625	1250	9370
Sitting on the bed ($A_2$)	5130	1285	705	7120
Sitting on the chair ($A_3$)	1255	310	1205	2770
Lying down on the bed ($A_4$)	12,870	3220	1540	17,630
Lying down on the floor ($A_5$)	9810	2455	3040	15,305
Empty room ($A_6$)	3405	850	640	4895
Transition ($A_7$)	4305	1075	790	6170
Total	43,270	10,820	9170	63,260

**Table 3:** Activity sample counts per subject in LOPO-CV approach. ($A_1$) walking, ($A_2$) sitting on the bed, ($A_3$) sitting on the chair, ($A_4$) lying down on the bed, ($A_5$) lying down on the floor, ($A_6$) empty room, ($A_7$) transition. ($S_1$) subject one, ($S_2$) subject two and ($S_3$) subject three.

Activity	$S_1$	$S_2$	$S_3$
$A_1$	3020	3800	2583
$A_2$	1800	2661	2684
$A_3$	1205	1568	–
$A_4$	5327	6142	6181
$A_5$	5993	6252	3082
$A_6$	1514	2030	1382
$A_7$	1749	2170	2298

To evaluate the models, we developed a normalization technique. Mean and standard deviation were computed from each feature channel using the combined training and validation data. These parameters normalized both the training-validation subset and the test set, ensuring uniform scaling and improving the learning process. All computations were performed on a high-performance system featuring an AMD Ryzen 7 6800H processor, 32 GB of system memory, and an NVIDIA GeForce RTX 3060 GPU running on Windows 11. For model development, we used Python 3.8.19, Scikit-learn 1.3.2, and TensorFlow 2.10. Table 4 summarizes the key hyperparameters, data augmentation settings, and system configuration used to train and evaluate the proposed lightweight model.

**Table 4:** Training configuration and hyperparameters used to train the proposed lightweight model.

Category	Setting
Optimizer	Stochastic gradient descent (SGD)
Initial learning rate	$1 \times 10^{-3}$
Learning-rate schedule	Fixed learning rate
Momentum	0.9
Weight decay	$1 \times 10^{-4}$
Batch size	8
Maximum epochs	300
Loss function	Categorical cross-entropy
Loss weighting	None (uniform class weighting)
Early stopping	Validation loss, patience = 30 epochs
Model selection	Best model saved based on minimum validation loss
Data augmentation	Spatial shifting ($p=0.8$); intensity scaling (0.9–1.1, $p=0.8$) and bias shift ($\pm 0.05$, $p=0.8$); horizontal Doppler flipping ($p=0.5$); additive Gaussian noise ($\mu =0$, $\sigma =0.02$, $p=0.8$)
Training hardware	AMD Ryzen 7 6800H CPU, 32 GB RAM, NVIDIA RTX 3060 GPU
Software framework	Python 3.8.19, TensorFlow 2.10, Scikit-learn 1.3.2

For comprehensive model evaluation, we employed different metrics for each validation approach. In the CSV strategy, we evaluated model performance through accuracy, precision, recall, and $F_1$-score. For the LOPO-CV approach, we calculated accuracy and $F_1$-score during classification of different numbers of activity of each subject, ultimately computing the average performance across all three subjects.

To further evaluate the performance of the proposed framework across varying classification complexities, four evaluation settings were defined according to the number of activity classes: seven-class, six-class, five-class, and four-class configurations. The full seven-class configuration included all recorded activities–Walking, Sitting on the Bed, Sitting on the Chair, Lying Down on the Bed, Lying Down on the Floor, Empty Room, and Transition. The six-class configuration excluded the “Transition” activity, which often involves intermediate motions between other activities and introduces ambiguity in radar signatures. The five-class configuration further excluded the “Empty Room” condition, as it primarily represents background radar noise rather than human motion. Finally, the four-class configuration focused on distinct posture- and motion-related activities–Walking, Sitting on the Bed, Sitting on the Chair, and Lying Down on the Bed–to emphasize recognition among the most representative human behaviors. This progressive reduction in the number of activity classes provides a systematic basis for assessing model robustness and generalization across increasingly simplified classification tasks.

### Experimental results

#### Cross-scene validation (CSV)

Table 5 compares the performance of the proposed framework with baseline ML (SVM, MLP) and DL (CNN, BiLSTM, ConvLSTM) models across multiple activity categories using combined RD, RA, and RE (RD+RA+RE) feature maps as input. The proposed model achieves the highest accuracy, with an overall performance of $91.98\%$ accuracy and an $F_1$-score of $89.82\%$ for seven activity classes, and an accuracy of $98.62\%$ and $F_1$-score of $98.70\%$ for four activity classes.

**Fig. 10:**
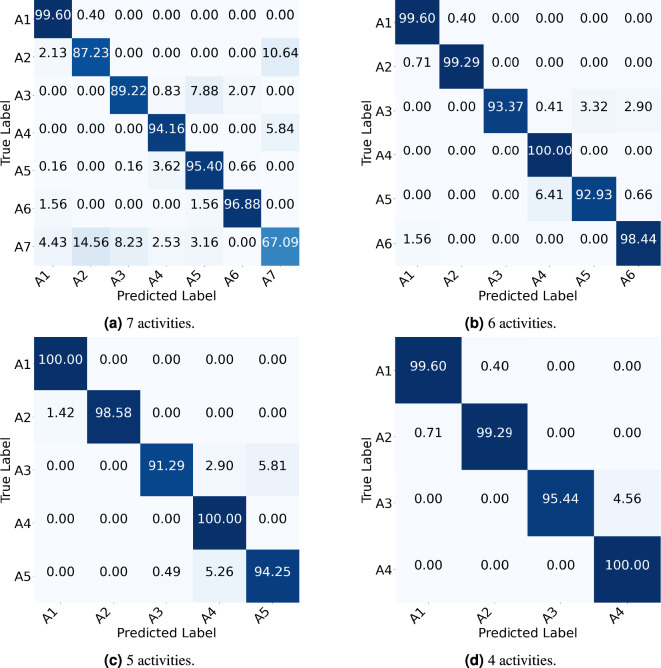
Confusion matrices of the proposed model evaluated under the CSV approach using RD+RA+RE feature inputs. ($A_1$) walking, ($A_2$) sitting on the bed, ($A_3$) sitting on the chair, ($A_4$) lying down on the bed, ($A_5$) lying down on the floor, ($A_6$) empty room, ($A_7$) transition.

The classification results of the proposed model across different feature inputs and varying numbers of activities are summarized in Tables 6, 7, 8 and 9. Additionally, Fig. 10 presents the confusion matrices for various activity sets using RD+RA+RE feature inputs.

As shown in Tables 6, 7, 8 and 9, classification performance improves as the number of classes decreases. Specifically, the $F_1$-score increases from $89.82\%$ for seven classes to $98.70\%$ for four classes. The RE map consistently achieves the best results across all tasks among single-feature inputs. For pairwise combinations, except for four classes, RD+RE maps outperform other combinations. Integrating all three features yields the highest performance, slightly surpassing RD+RE maps. This highlights the significance of combining motion (Doppler) and spatial dimensions (azimuth and elevation) to comprehensively represent activities, thereby enhancing classification accuracy.

Table 10 provides a comprehensive comparison of the proposed model’s performance when trained with and without proposed data augmentation using RD+RA+RE feature maps. The results clearly demonstrate that applying augmentation consistently improves all key metrics across every activity classification task, from seven-class to four-class problems. For instance, in the 7-activity scenario, augmentation boosts the accuracy from 84.90% to 91.98% and the $F_1$-score from 83.18% to 89.82%. Similar trends are observed for 6, 5, and 4 activities, where both overall performance and class-wise discrimination benefit from augmented training data. These improvements highlight the crucial role of augmentation in enhancing the model’s ability to generalize, particularly in challenging, multi-class human activity recognition tasks.

**Fig. 11:**
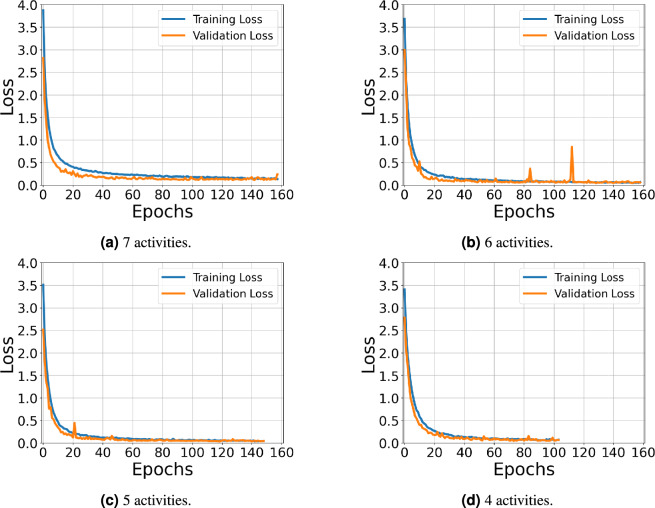
Training and validation loss curves for the proposed model under the CSV approach using RD+RA+RE feature inputs.

Figure 11 visualizes the training and validation loss curves of the proposed model across different activity sets using RD+RA+RE feature inputs. The training progression shows a consistent decline in both training and validation losses, reaching satisfactorily low values, indicating effective model learning, the absence of overfitting, and robust generalization.

**Table 5:** Comparative performance of different ML and DL models under the CSV approach using RD+RA+RE feature inputs.

#Activities	Model	Metrics (%)			
		Accuracy	Precision	Recall	$F_1$-score
7	SVM	70.97	72.20	74.17	70.41
	MLP	68.71	66.35	68.76	65.85
	CNN	89.48	86.66	88.81	87.17
	BiLSTM	88.50	85.09	87.35	85.64
	ConvLSTM	90.51	86.75	88.51	87.31
	Proposed model	**91.98**	**89.85**	**89.93**	**89.82**
6	SVM	76.34	80.11	82.02	77.75
	MLP	75.40	76.30	78.77	75.58
	CNN	92.60	91.59	93.67	91.83
	BiLSTM	94.03	92.87	95.19	93.55
	ConvLSTM	95.29	94.08	96.02	94.73
	Proposed model	**96.24**	**96.19**	**97.26**	**96.62**
5	SVM	76.63	83.76	81.19	79.86
	MLP	78.25	82.23	83.22	80.99
	CNN	92.57	94.09	94.40	93.79
	BiLSTM	93.99	95.08	95.08	94.79
	ConvLSTM	96.06	**96.86**	96.15	96.39
	Proposed model	**96.25**	96.84	**96.82**	**96.73**
4	SVM	88.80	92.16	88.53	89.08
	MLP	90.28	92.26	90.33	90.43
	CNN	96.28	97.12	96.17	96.49
	BiLSTM	96.91	97.61	96.79	97.11
	ConvLSTM	97.87	98.39	97.80	98.05
	Proposed model	**98.62**	**98.86**	**98.58**	**98.70**

Significant values are in bold.

**Table 6:** Comparison of proposed model performance for 7 activity classification under the CSV approach.

Metrics (%)	Model input						
	RD	RA	RE	RD+RA	RD+RE	RA+RE	RD+RA+RE
Accuracy	74.54	76.06	88.28	83.75	90.51	85.11	91.98
Precision	79.97	76.34	84.38	83.90	88.66	82.38	89.85
Recall	78.90	80.45	87.36	85.54	89.35	85.26	89.93
$F_1$-score	77.30	76.32	85.37	84.00	88.47	82.98	89.82

**Table 7:** Comparison of proposed model performance for 6 activity classification under the CSV approach.

Metrics (%)	Model input						
	RD	RA	RE	RD+RA	RD+RE	RA+RE	RD+RA+RE
Accuracy	77.68	80.61	93.62	91.11	94.21	91.71	96.24
Precision	82.59	84.26	92.45	92.87	94.36	91.10	96.19
Recall	83.67	87.64	94.29	94.23	95.25	93.72	97.26
$F_1$-score	81.19	83.41	92.89	93.09	94.57	91.71	96.62

**Table 8:** Comparison of proposed model performance for 5 activity classification under the CSV approach.

Metrics (%)	Model input						
	RD	RA	RE	RD+RA	RD+RE	RA+RE	RD+RA+RE
Accuracy	73.71	81.27	94.57	85.34	94.70	94.06	96.25
Precision	82.54	86.99	94.98	89.90	95.32	95.27	96.84
Recall	81.64	88.22	95.73	89.79	96.09	95.24	96.82
$F_1$-score	78.44	85.72	95.23	88.55	95.52	95.04	96.73

**Table 9:** Comparison of proposed model performance for 4 activity classification under the CSV approach.

Metrics (%)	Model Input						
	RD	RA	RE	RD+RA	RD+RE	RA+RE	RD+RA+RE
Accuracy	85.32	96.38	98.51	97.34	96.81	98.30	98.62
Precision	86.26	96.81	98.68	97.76	97.52	98.64	98.86
Recall	85.81	96.29	98.40	97.43	96.67	97.97	98.58
$F_1$-score	83.98	96.47	98.53	97.52	96.98	98.28	98.70

**Table 10:** Performance comparison of the proposed model with and without data augmentation using RD+RA+RE feature inputs under the CSV approach.

#Activities	Model	Metrics (%)			
		Accuracy	Precision	Recall	$F_1$-score
7	Without augmentation	84.90	83.25	84.44	83.18
	With augmentation	**91.98**	**89.85**	**89.93**	**89.82**
6	Without augmentation	94.15	94.41	95.63	94.77
	With augmentation	**96.24**	**96.19**	**97.26**	**96.62**
5	Without augmentation	95.74	96.12	**96.90**	96.39
	With augmentation	**96.25**	**96.84**	96.82	**96.73**
4	Without augmentation	97.87	98.44	97.70	98.02
	With augmentation	**98.62**	**98.86**	**98.58**	**98.70**

Significant values are in bold.

#### Leave-one-person-out cross-validation (LOPO-CV)

We report LOPO-CV results for the proposed model using RD+RA+RE feature maps, which outperformed all baseline models in the CSV evaluation setting. Table 11 presents the performance of the proposed model in activity classification, achieving an overall accuracy of $89.74\%$ and an $F_1$-score of $83.24\%$ for seven activities. When the number of activity classes was reduced to four, performance improved to $94.47\%$ accuracy and an $F_1$-score of $85.86\%$. These results confirm the model’s subject-independent robustness, generalizability, and the effectiveness of RD+RA+RE feature maps. Compared to the CSV approach, this method evaluates the model on a larger test set.

From Table 11, it is evident that the model performance decreases noticeably when tested on Subject 3, particularly in terms of the $F_1$-score. This reduction can primarily be attributed to data imbalance and incomplete activity coverage for this subject, as shown in Table 3. Specifically, Subject 3 lacks samples for one activity class ($A_3$: Sitting on the Chair), reducing the diversity of radar signatures available for model evaluation and resulting in lower recall and, consequently, a reduced $F_1$-score. These observations indicate that the model’s performance is influenced by both the quantity and class distribution of subject-specific data, rather than by limitations in the model architecture itself. Notably, performance analysis of the proposed model with single- and pairwise-feature map combinations was not conducted within the LOPO-CV protocol.

**Table 11:** Comparison of proposed model performance presented as percentages under LOPO-CV with RD+RA+RE inputs.

Test subject	7 activity		6 activity		5 activity		4 activity	
	Accuracy	$F_1$-score	Accuracy	$F_1$-score	Accuracy	$F_1$-score	Accuracy	$F_1$-score
Subject 1	87.99	83.83	94.69	93.62	94.63	93.90	94.09	92.95
Subject 2	94.44	92.76	97.59	97.11	98.45	98.25	98.87	98.67
Subject 3	86.78	73.12	91.96	91.36	89.34	69.20	90.46	65.95
Average	89.74	83.24	94.75	94.03	94.14	87.12	94.47	85.86

**Fig. 12:**
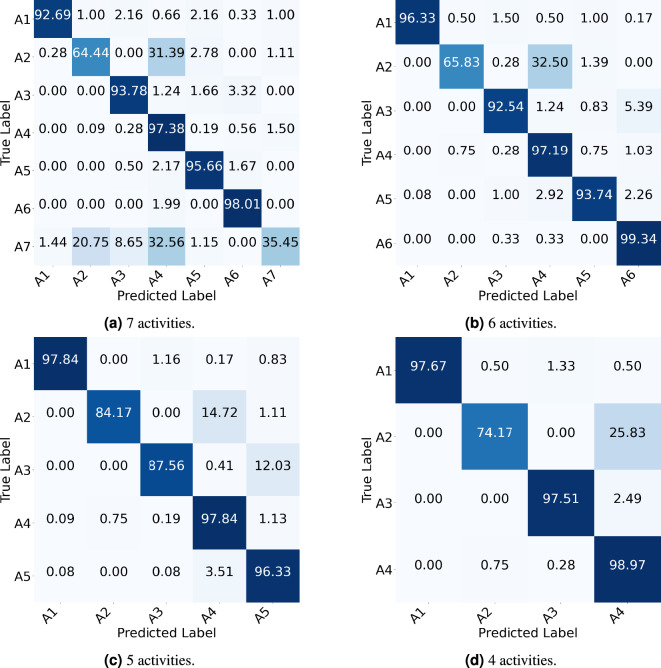
Confusion matrices of the proposed model evaluated under the CSV approach using combined RD+RA+RE feature inputs, trained on data from a single subject and tested on an unseen participant. ($A_1$) walking, ($A_2$) sitting on the bed, ($A_3$) sitting on the chair, ($A_4$) lying down on the bed, ($A_5$) lying down on the floor, ($A_6$) empty room, ($A_7$) transition.

**Fig. 13:**
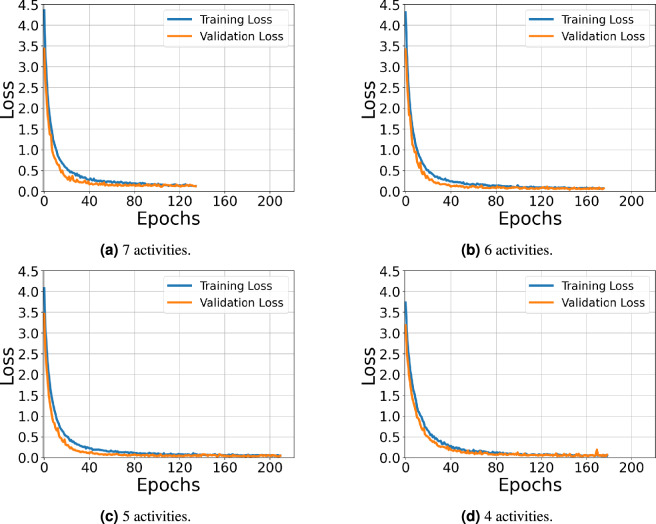
Training and validation loss curves of the proposed model under the CSV approach using combined RD+RA+RE feature inputs, trained on data from a single subject and tested on an unseen participant.

To further evaluate the robustness of the proposed model under limited training data, an additional experiment was conducted in which the model was trained using RD+RA+RE feature maps from a single subject (subject 2) and tested on a different, unseen participant (subject 1) across varying activity sets (Table 12). Despite the minimal training data, the model maintained strong performance, demonstrating its ability to handle challenging scenarios with highly restricted datasets. Figure 12 presents the confusion matrices for different activity sets using RD+RA+RE feature inputs, while Fig. 13 shows the corresponding training and validation loss curves of the proposed model.

**Table 12:** Evaluation of the proposed model trained on data from a single subject and tested on an unseen participant across varying activity sets.

#Activities	Metrics (%)			
	Accuracy	Precision	Recall	$F_1$-score
7	87.91	86.19	82.48	82.71
6	92.83	92.49	90.82	91.05
5	95.18	95.97	92.74	94.21
4	94.53	95.68	92.07	93.49

To facilitate a clear comparison of the proposed model under the two validation strategies, Table 13 summarizes the activity classification results for different numbers of activities using CSV and LOPO-CV. For LOPO-CV, the reported values are presented as mean ± standard deviation across three folds. In contrast, due to the limited number of available scenes, CSV is evaluated using a fixed cross-scene split; therefore, only the best performance value is reported.

**Table 13:** Activity classification performance (%) using RD+RA+RE features under cross-scene validation (CSV) and leave-one-person-out cross-validation (LOPO-CV) for 4–7 activities. LOPO-CV results are reported as mean ± standard deviation across three folds, while CSV reports the best result from a fixed cross-scene split.

Validation	7 activity		6 activity		5 activity		4 activity	
	Accuracy	$F_1$-score	Accuracy	$F_1$-score	Accuracy	$F_1$-score	Accuracy	$F_1$-score
CSV	91.98	89.82	96.24	96.62	96.25	96.73	98.62	98.70
LOPO-CV	89.74 ± 4.12	83.24 ± 9.83	94.75 ± 2.82	94.03 ± 2.90	94.14 ± 4.57	87.12 ± 15.67	94.47 ± 4.22	85.86 ± 17.48

#### Computational efficiency

Table 14 presents a comparative analysis of the computational complexity of the models evaluated in this study, highlighting the number of parameters (in millions—M), floating-point operations per second (FLOPs) measured in GigaFLOPS (GFLOPS), and model size (in megabytes—MB). The results reveal notable differences in computational cost among the models. The BiLSTM, while providing strong temporal modeling, exhibits the highest number of parameters (102.76M) and the largest model size (1204.38 MB), which restricts its suitability for resource-constrained environments. ConvLSTM, though delivering improved performance for activity recognition, also imposes a considerable computational load (41.98M parameters, 328 MB). In contrast, the proposed model offers a highly favorable trade-off, achieving competitive recognition accuracy with minimal complexity (0.814M parameters, 6.66 MB). Its reduced FLOPs (0.0696 GFLOPS) confirm its efficiency during inference, supporting its suitability for real-time applications and large-scale deployment, such as in care centers or hospitals. These findings demonstrate that the proposed model requires minimal computational resources and underscore the importance of model optimization for activity recognition, particularly where deployment on devices with limited computational power and memory is essential.

**Table 14:** Comparison of model complexity in terms of parameters, FLOPs, and model size for all evaluated architectures.

Model	#Parameter (M)	#Flops (GFLOPS)	Size (MB)
CNN	2.7	0.5373	31.70
BiLSTM	102.76	0.0005	1204.38
ConvLSTM	41.98	0.1438	328
Proposed model	0.814	0.0696	6.66

Although the proposed framework is significantly smaller than the compared baselines, it achieves comparable performance to the other evaluated deep learning models.

## Discussion

The results of this study clearly demonstrate the effectiveness of the proposed lightweight FMCW radar-based framework for HAR. By integrating RD, RA, and RE feature maps, the system was comprehensively validated under two distinct protocols: CSV and LOPO-CV.

Across both evaluation strategies, the proposed ResNet-18 + BiLSTM model consistently outperformed all baseline ML (SVM, MLP) and DL (CNN, BiLSTM, ConvLSTM) approaches. Under the CSV protocol, it achieved an accuracy of $91.98\%$ and an $F_1$-score of $89.82\%$ for seven activities, surpassing the closest ConvLSTM baseline by more than one percentage point for both metrics. The model also performed well under LOPO-CV, achieving $89.74\%$ accuracy and an $F_1$-score of $83.24\%$, confirming its robust generalization across unseen participants and environments. The smooth convergence of training and validation loss curves (Fig. 11) indicates stable optimization and minimal overfitting.

A comparative evaluation of different radar feature inputs under the CSV protocol further highlights the importance of combining complementary spatial and temporal information. As summarized in Tables 6, 7, 8, 9, models trained with individual feature maps (RD, RA, or RE) exhibited noticeably lower accuracy compared to those using combined inputs. Among single-feature inputs, RE yielded the highest recognition accuracy, underscoring the importance of vertical spatial cues. Pairwise combinations such as RD+RA and RD+RE provided intermediate improvements, while the full combination of RD+RA+RE consistently achieved the best results across all activity configurations. These findings empirically confirm that multi-dimensional feature fusion enables richer motion representation and enhances model robustness in complex activity scenarios.

The superior performance of the proposed model can be attributed to the integration of SeparableConv2D and BiLSTM layers, which facilitate efficient spatial-temporal representation learning in a lightweight architecture. The ResNet backbone enhances gradient stability through residual connections, while the BiLSTM module captures bidirectional temporal dependencies across sequential radar frames. Together, these design components provide an optimal balance between recognition accuracy and computational efficiency, as evidenced by the model’s low parameter count (0.814M) and compact size (6.66 MB), enabling real-time deployment on edge devices.

In addition, the proposed lightweight framework’s ability to maintain, and in some cases surpass, the accuracy of significantly heavier architectures can be attributed to the alignment between the model’s inductive bias and the physical properties of radar data. Unlike baseline models that rely on dense connections, the use of depthwise separable convolutions decouples spatial filtering and channel-wise feature combination by factorizing a standard convolution into a depthwise convolution and a $1 \times 1$ pointwise convolution^46^. This design has been shown to “drastically reduce computation and model size”^46^, enabling compact architectures that remain effective while being more suitable for latency- and resource-constrained deployment. Furthermore, in the context of limited training data, large-capacity models are more prone to overfitting, since “with limited training data” some learned relationships may reflect “sampling noise,” which can lead to poor generalization^41^. In contrast, the compact nature of the proposed model can help reduce this risk by limiting unnecessary model capacity and encouraging the learning of more robust motion patterns. Finally, by processing structured 3D data vectors directly, the model preserves the physical meaning of the radar axes and avoids additional image-generation or resizing steps, allowing the available model capacity to focus on physically meaningful feature extraction.

A key contribution of this work is the direct use of multi-dimensional radar feature maps (RD, RA, and RE) as structured data vectors, instead of converting them into image representations. This design reduces preprocessing overhead and preserves the separable physical dimensions of the radar measurements, allowing the network to learn spatio-temporal motion patterns more effectively. As a result, the proposed framework achieves robust recognition performance even when trained on data from only three subjects, highlighting its suitability for practical scenarios where collecting large-scale radar datasets is challenging.

Another significant contribution is the development of a new radar-based HAR dataset collected in a realistic, home-like environment. Unlike most existing datasets acquired under controlled laboratory conditions, the proposed dataset reflects everyday settings and includes a broad spectrum of common and complex human activities. This diversity enhances ecological validity and supports more generalizable model development. To facilitate further research, the dataset will be available upon reasonable request as a benchmark resource for advanced analysis and future studies in radar-based HAR.

In addition, the effectiveness of the implemented data augmentation strategies–spatial shifting, temporal warping, intensity scaling, and additive noise–was quantitatively confirmed (Table 10). These methods led to substantial improvements in both accuracy and $F_1$-score, with the greatest gains observed in the most complex, seven-class recognition tasks. The augmentation strategies improve model robustness by simulating spatial misalignments, temporal distortions, and signal fluctuations typical in real-world radar data, thereby enhancing generalization under limited data conditions.

Overall, these findings advance radar-based HAR by demonstrating that high recognition accuracy and generalizability can be attained using a compact, computationally efficient model trained on limited data. The results validate the joint effect of targeted augmentation, structured feature representation, and lightweight architecture on enhancing model robustness and adaptability to unseen scenarios.

Despite these promising results, certain limitations persist. The dataset, while diverse, includes a limited number of participants and types of activities. Moreover, further analysis of computational complexity and inference latency is needed to quantify real-time performance on edge devices rigorously. Future work will address these limitations by expanding dataset diversity and investigate adaptive or transfer learning approaches to enhance scalability and cross-domain generalization.

From an application perspective, the proposed framework offers a promising foundation for unobtrusive and privacy-preserving human activity monitoring. Its lightweight design and edge-optimized processing make it ideal for deployment in smart homes, healthcare settings, and eldercare environments, where real-time operation and low power consumption are critical. This study illustrates that efficient model design, realistic data collection, and well-chosen augmentation strategies can collectively bridge the gap between high performance and practical deployability in HAR systems.

## Conclusion

In this paper, we proposed a lightweight framework for HAR using FMCW radar, designed and validated in a realistic, home-like environment. This study demonstrated the feasibility and advantages of leveraging multi-dimensional radar feature maps—RD, RA, and RE—as data vectors to capture the spatial and temporal characteristics of human activities effectively. By integrating these feature representations within a lightweight model architecture, the proposed framework achieved superior performance compared to baseline ML and DL approaches, confirming its robustness and generalization capability.

The results further highlight the benefits of combining multiple radar feature maps, with RE information proving especially valuable for improving recognition accuracy, due to its inclusion of vertical spatial cues. The integration of SeparableConv2D and BiLSTM layers enabled efficient spatial-temporal modeling, while maintaining a compact network optimized for edge computing. Additionally, the data augmentation strategies implemented in this work proved effective for improving generalization.

A notable contribution of this study is the introduction of a new FMCW radar dataset collected in a realistic, home-like environment that encompasses a diverse array of everyday activities. Unlike most existing laboratory-based datasets, this resource captures complex and naturally occurring motions, thereby increasing ecological validity. To promote future research and facilitate reproducibility, the dataset will be publicly available upon publication as a benchmark resource for radar-based HAR studies.

In summary, this study presents a novel and efficient FMCW radar-based framework for unobtrusive human activity recognition, combining lightweight model design, realistic data collection, and effective augmentation strategies. The findings demonstrate that high recognition accuracy and real-world applicability can be achieved even with limited data, paving the way for scalable, privacy-preserving, and edge-deployable activity-monitoring systems across applications such as healthcare, rehabilitation, and smart home environments.

## Data Availability

The data supporting the conclusions of this article will be made available by the corresponding author, S.Z., upon request, without undue reservation.
